# Predicting the potential distribution of the parasitic *Cuscuta chinensis* under global warming

**DOI:** 10.1186/s12898-020-00295-6

**Published:** 2020-05-09

**Authors:** Zichun Ren, Lyuben Zagortchev, Junxia Ma, Ming Yan, Junmin Li

**Affiliations:** 1 School of Life Science, Shanxi Normal University, Linfen, 041000 China; 2 Zhejiang Provincial Key Laboratory of Plant Evolutionary Ecology and Conservation, Taizhou University, Taizhou, 318000 China; 3 Department of Biochemistry, Faculty of Biology, Sofia University “St. Kliment Ohridski”, 8 Dragan Tsankovblvd., 1164 Sofia, Bulgaria

**Keywords:** *Cuscuta chinensis*, Ecological niche model, Maxent model, Bioclimatic variables, Species distribution, Climatic warming

## Abstract

**Background:**

The climate is the dominant factor that affects the distribution of plants. *Cuscuta chinensis* is a stem holoparasitic plant without leaves or roots, which develops a haustorium and sucks nutrients from host plants. The potential distribution of the parasitic plant *C. chinensis* has not been predicted to date. This study used Maxent modeling to predict the potential global distribution of *C. chinensis*, based on the following six main bioclimatic variables: annual mean temperature, isothermality, temperature seasonality, precipitation seasonality, precipitation of the warmest quarter, and precipitation of the coldest quarter.

**Results:**

The optimal annual average temperature and isothermality of *C. chinensis* ranged from 4 to 37 °C and less than 45, respectively, while the optimal temperature seasonality and precipitation seasonality ranged from 4000 to 25,000 and from 50 to 130, respectively. The optimal precipitation of the warmest season ranged from 300 to 1000 mm and from 2500 to 3500 mm, while that of the coldest season was less than 2000 mm. In Asia, *C. chinensis* is mainly distributed at latitudes ranging from 20° N to 50° N. During three specific historical periods (last glacial maximum, mid-Holocene, and 1960–1990) the habitats suitable for *C. chinensis* were concentrated in the central, northern, southern, and eastern parts of China. From the last glacial maximum to the mid-Holocene, the total area with suitability of 0.5–1 increased by 0.0875 million km^2^; however, from the mid-Holocene to 1960–1990, the total area with suitability of 0.5–1 decreased by 0.0759 million km^2^. The simulation results of habitat suitability in the two representative concentration pathways (RCP) 2.6 (i.e., the low greenhouse gas emissions pathway) and 8.5 (i.e., the high greenhouse gas emissions pathway) indicate that the habitat suitability of *C. chinensis* decreased in response to the warming climate. Compared with RCP2.6, areas with averaged suitability and high suitability for survival (RCP8.5) decreased by 0.18 million km^2^.

**Conclusion:**

Suitable habitats of *C. chinensis* are situated in central, northern, southern, and eastern China. The habitat suitability of *C. chinensis* decreased in response to the warming climate. These results provide a reference for the management and control of *C. chinensis*.

## Background

The climate is the dominant factor to affect plant distribution [1–4]. Under the context of global climate change, increasing attention has been focused on the prediction of the distribution of plants to better apprehend future trends [4]. In addition to species distributed in unique habitats, such as arid regions [5], highlands [4], and capes [6], most studies focused on specific species, such as dominant forest species [7, 8], invasive species [9, 10], or rare species [3] to enable better management of species. However, to date, only few studies focused on parasitic plants [11, 12].

By definition, parasitic plants obtain all or part of their energy from autotrophic plants (producers) via haustaria, and are ubiquitous species in all ecosystems [13]. Most of the parasitic plants are harmful to agriculture since they absorb a notable share of the host plants’ nutrients, which ultimately decreases or even inhibits host growth and can lead to the death of the host due to insufficient nutrition [14]. However, many parasitic plants are also used as medicine, such as *Cuscuta chinensis* [15], *Cistanche deserticola* [16], and *Viscum coloratum* [17]. Predicting the potential global distribution of such parasitic plants not only informs management procedures that enable a reduction of the harm parasitic species impose on agriculture, it is also useful for the development of medical applications. Until now, the distribution of *C. chinensis* has not been predicted.

Ecological niche models (ENMs) are standard in ecological modelling [18]. ENMs are a classical method that utilizes occurrence data in conjunction with environmental data to build a correlative model of the specific environmental conditions that meet a species’ ecological requirements and thus predict the relative habitat suitability [19]. ENMs use layers of environmental information and species, as well as pseudo-absence or absence points to develop probabilistic maps of suitable distribution [20]. ENMs are generally used for four main objectives: (1) to estimate the relative suitability of the habitat that is currently occupied by the species to be assessed, (2) to estimate the relative habitat suitability in areas where the assessed species are currently not known to be present, (3) to estimate potential changes in the habitat suitability due to scenarios of environmental change, and (4) to estimate the environmental niche of a species [21]. Among the available tools for ENMs, the maximum entropy (Maxent) approach is one of the most widely used for the prediction of species distributions [20, 22]. Moreover, Maxent is effective for the prediction of narrow species distributions [2, 3, 23–27].

*Cuscuta* spp. belong to the family of Convolvulaceae and are annual holoparasitic herbs. *Cuscuta* spp. grow in a wide variety of climates and ecosystems on all continents except Antarctica [28]. *Cuscuta* spp. severely damage crop plants and are considered as the third-most detrimental group of parasitic pants worldwide following *Striga* and *Orobance* [28]. *C. chinensis* is a typical native holoparasitic plant belonged to *Cuscuta* genus in China, which is also known as the Chinese Dodder [29], or Tu-Si-Zi in Chinese [30]. This study used the Maxent method to predict the potential distribution of *C. chinensis* based on world-wide occurrence data of *C. chinensis*. This study aimed to identify: (1) the climatic factors that affect the suitability of *C. chinensis* habitat,( 2) how the distribution of *C. chinensis* changed during three historical periods (last glacial maximum, mid-Holocene, and 1960–1990), and (3) how the *C. chinensis* distribution changed in response to global warming. The results provide a basic understanding of the trends of parasitic *Cuscuta* spp. plants within the plant community and improve the management and control of this species.

## Results

### Model performance and contribution of variables

Ecological modeling yielded an average AUC value of 0.951, while the TSS index was 0.887, classifying the model as very satisfactory. The six bioclimatic variables of annual mean temperature (Bio1), isothermality (Bio3), temperature seasonality (Bio4), precipitation seasonality (Bio15), precipitation of warmest quarter (Bio18), and precipitation of coldest quarter (Bio19) were selected to establish the model (Table 1). Additional file 1: Fig. S1 shows the results of the Jackknife test of the variable contribution by Maxent. When used independently, Bio1, Bio3, Bio15, and Bio18 provided very high gains (> 0.40), indicating that these four variables contained more useful information than the other variables. Bio4 and Bio19 achieved very low yields when used alone, and did not contain much information. Therefore, Bio1, Bio3, Bio15, and Bio18 were identified as important climatic factors that influence the suitable habitat of *C. chinensis*.

**Table 1:** Environmental variables used for modeling the habitat suitability distribution of *C. chinensis* in this study

Data source	Category	Variables	Abbreviation	Units
*C. chinensis* Worldclime	Bioclimatic	Annual mean temperature	Bio1	°C
		Isothermality (BIO2/BIO7) (*100)	Bio3	Dimensionless
		Temperature seasonality (standard deviation *100)	Bio4	Dimensionless
		Precipitation seasonality (coefficient of variation)	Bio15	Dimensionless
		Precipitation of warmest quarter	Bio18	mm
		Precipitation of coldest quarter	Bio19	mm

### Response of variables to suitability

The response curves of *C. chinensis* to the six assessed bioclimatic variables are shown in Additional file 2: Fig. S2. As shown in Additional file 2: Fig. S2a, when Bio1 is below 5 °C, the probability that *C. chinensis* exists is extremely low (below 0.5, indicating low probability). With increasing temperature, the probability for *C. chinensis* to exist gradually increased, and reached the maximum at 22 °C with a probability of existence as high as 0.7. When Bio1 ranges from 4 to 37 °C, the survival rate of *C. chinensis* was high (~ 0.5). Therefore, the optimum annual mean temperature of *C. chinensis* ranges from 4 °C to 37 °C.

As shown in Additional file 2: Fig. S2b, when Bio3 ranged from 0 to 45, the survival probability of *C. chinensis* exceeded 0.5, indicating a benefit for the survival of *C. chinensis*. Therefore, the optimum isothermality of *C. chinensis* should remain below 45.

As shown in Additional file 2: Fig. S2c, when the temperature seasonality of Bio4 was ~ 500 or less, the existence probability of *C. chinensis* was extremely low. Furthermore, from 4000 to 25,000, the survival probability of *C. chinensis* first increased and then decreased, and the survival probability of *C. chinensis* decreased to above 0.5. Therefore, the optimal temperature seasonality of *C. chinensis* is 4000–25,000.

As shown in Additional file 2: Fig. S2d, when Bio15 exceeds 25, the survival probability of *C. chinensis* rapidly increased and reached a peaked at around 80 (~ 0.72). From 50 to 130, the survival probability of *C. chinensis* exceeded 0.5. Therefore, the optimal precipitation seasonality ranges from 50 to 130.

As shown in Additional file 2: Fig. S2e, with increasing Bio18, the survival probability of *C. chinensis* gradually increased and peaked at around 500 mm. Beyond 500 mm, the survival probability of *C. chinensis* deceased and reached a minimum at around 1400 mm. Furthermore, the optimal precipitation of the warmest quarter ranges from 300 to 1000 mm, and from 2500 to 3500 mm with the survival probability of *C. chinensis* exceeding 0.5.

As shown in Additional file 2: Fig. S2f, when Bio19 ranged from 0 to 2000 mm, the existence probability of *C. chinensis* exceeded 0.5. Therefore, the optimal precipitation of *C. chinensis* in the coldest season ranges from 0 mm to 2000 mm and the survival probability exceeds 0.5.

### Model application

#### Global *C. chinensis* distribution

**Fig. 1:**
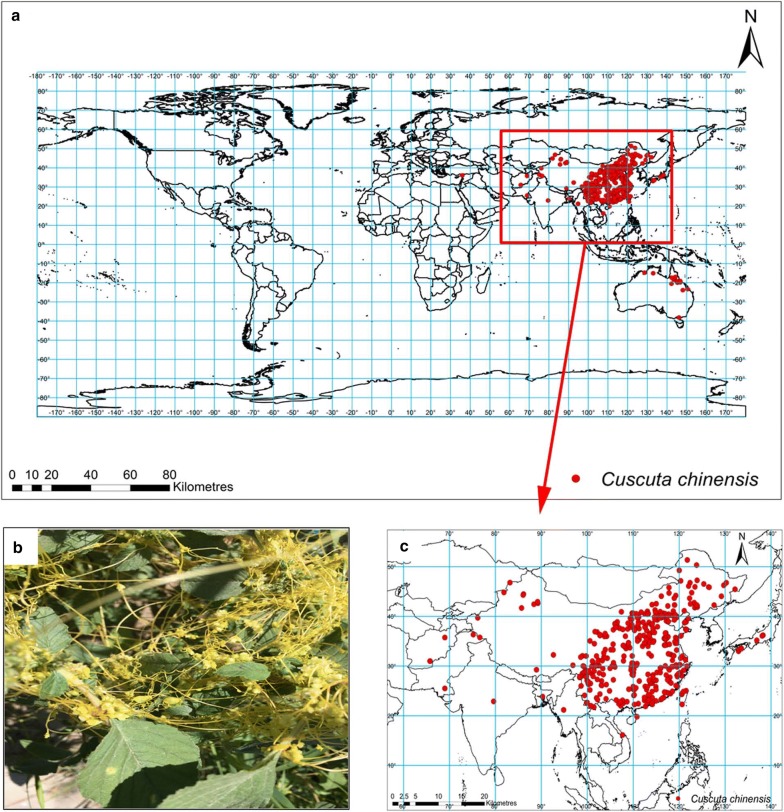
Global distribution (**a**), photo (**b**) and concentrated distribution (**c**) of *Cuscuta chinensis*. Geographical base map data were downloaded from the national basic geographic information system (http://www.diva-gis.org/)

The global *C. chinensis* distribution is shown in Fig. 1a. In Asia, *C. chinensis* are mainly distributed at latitudes ranging from 20° N to 50° N, which includes central, eastern, and southern China (Fig. 1c). *C. chinensis* also has a small distribution in Japan, India, Afghanistan, Pakistan, Myanmar, Vietnam, Bangladesh, and Turkey as well as minor occurrences in Australia (Fig. 1a, c). However, no distribution was found on Europe, Africa, and America (Fig. 1a).

#### Habitat suitability simulation for three historical periods

**Fig. 2:**
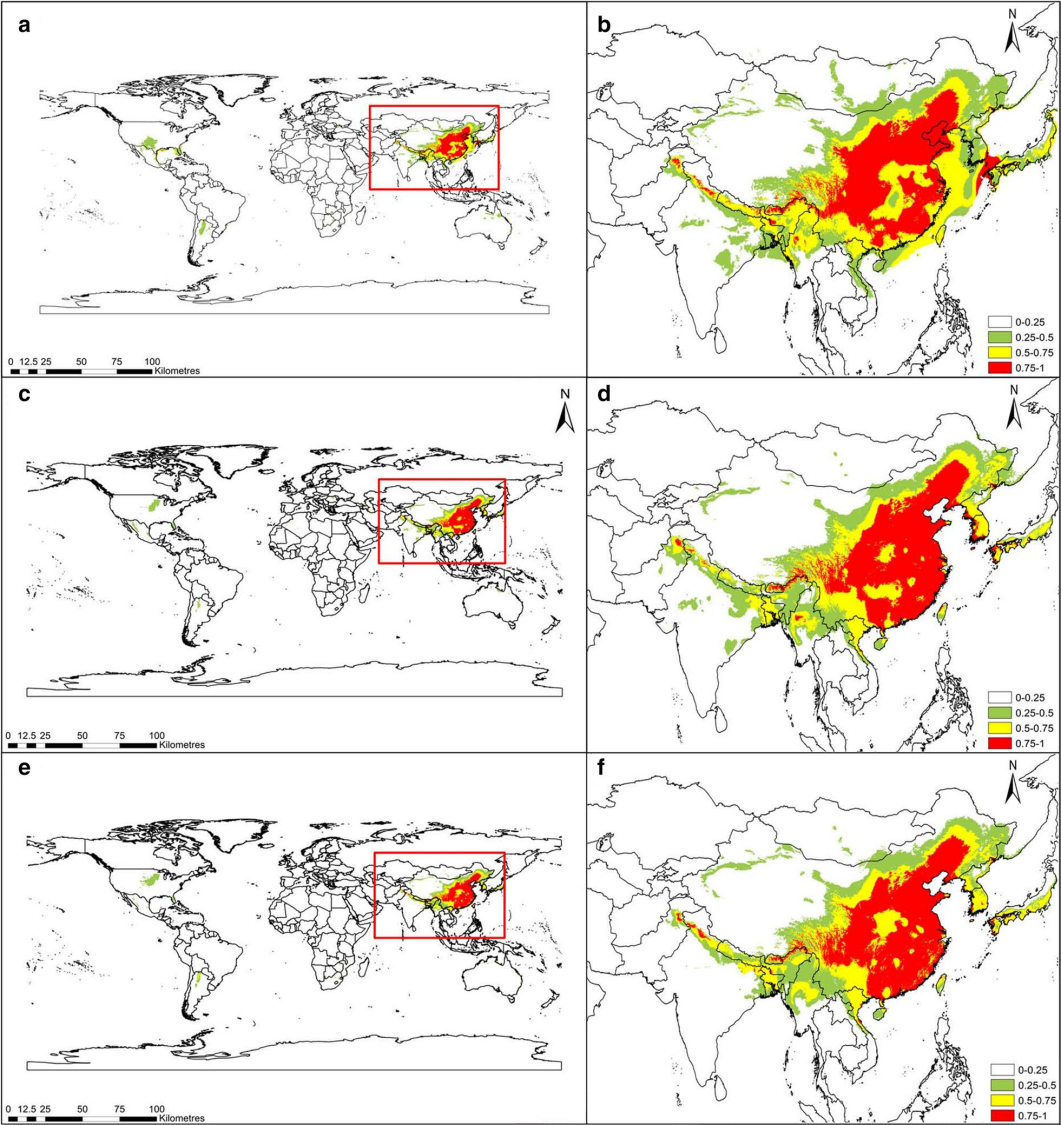
Habitat suitability distribution of *Cuscuta chinensis* for three historical periods. Last glacial maximum (**a**, **b**), mid-Holocene (**c**, **d**), and 1960–1990 (**e**, **f**). Geographical base map data were downloaded from the national basic geographic information system (http://www.diva-gis.org/)

The simulation results of the *C. chinensis* habitat suitability during three historical periods (last glacial maximum, mid-Holocene, and 1960–1990) are shown in Fig. 2. From the perspective of space, suitable areas for *C. chinensis* during these three periods concentrated in the central, northern, southern, and eastern parts of China. These areas have a survival probability above 0.5, indicating that *C. chinensis* in these region benefitted from moderate or relatively high suitability. Compared with the last glacial maximum, the paleoclimatic prediction of the Holocene mid-term CCSM4 climate model indicates that the position in the mid-Holocene changed; moreover, it indicated that the size of the predicted distribution increased. From the mid-Holocene to 1960–1990, the global habitat suitability of *C. chinensis* gradually decreased, and the area with medium and relatively higher fitness (> 0.5) gradually decreased. From the last glacial maximum to the mid-Holocene, the total area with suitability above 0.75 increased by 0.5689 million km^2^ (i.e., by 25.09%). The area with higher fitness (> 0.75) during the mid-Holocene reached 2.8362 million km^2^, accounting for 1.9% of the global total area. However, from the mid-Holocene to 1960–1990, the total area with suitability above 0.75 decreased by 0.0797 million km^2^ (i.e., by 2.81%). During the period of 1960-1990, the area with high fitness (> 0.75) was 2.7565 million km^2^, accounting for 1.85% of the global total area. From the last glacial maximum to the mid-Holocene, the total area with suitability of 0.5-1 increased by 0.0875 million km^2^, while from the mid-Holocene to 1960–1990, the total area with suitability of 0.5–1 decreased by 0.0759 million km^2^ (Table 2).

**Table 2:** The area of *Cuscuta chinensis*’s four habitat suitability distribution during three historical periods

Habitat suitability	Historical periods								
	Last glacial maximum			Mid-holocene			1960–1990		
	Area (million km^2^)	TAPCT (%)	PCT (%)	Area (million km^2^)	TAPCT (%)	PCT (%)	Area (million km^2^)	TAPCT (%)	PCT (%)
0–0.25	140.0203	–	94.04	140.6176	+ 0.43	94.44	140.7554	+ 0.01	94.53
0.25–0.5	4.0135	–	2.70	3.3286	− 17.06	2.24	3.2669	− 1.86	2.19
0.5–0.75	2.5989	–	1.75	2.1175	− 18.52	1.42	2.1213	+ 0.18	1.42
0.75–1	2.2673	–	1.52	2.8362	+ 25.09	1.90	2.7565	− 2.81	1.85

PCT indicates the percentage of the area in the current historical periods of the global’s total area. TAPCT indicates the percentage of the area in the current historical periods relative to the area in the last historical periods. The percentage increase (“+”) and decrease (“−”) in the area of the column compared to the area of the previous column in the historical period

#### Suitable habitat distributions under global warming scenarios

**Fig. 3:**
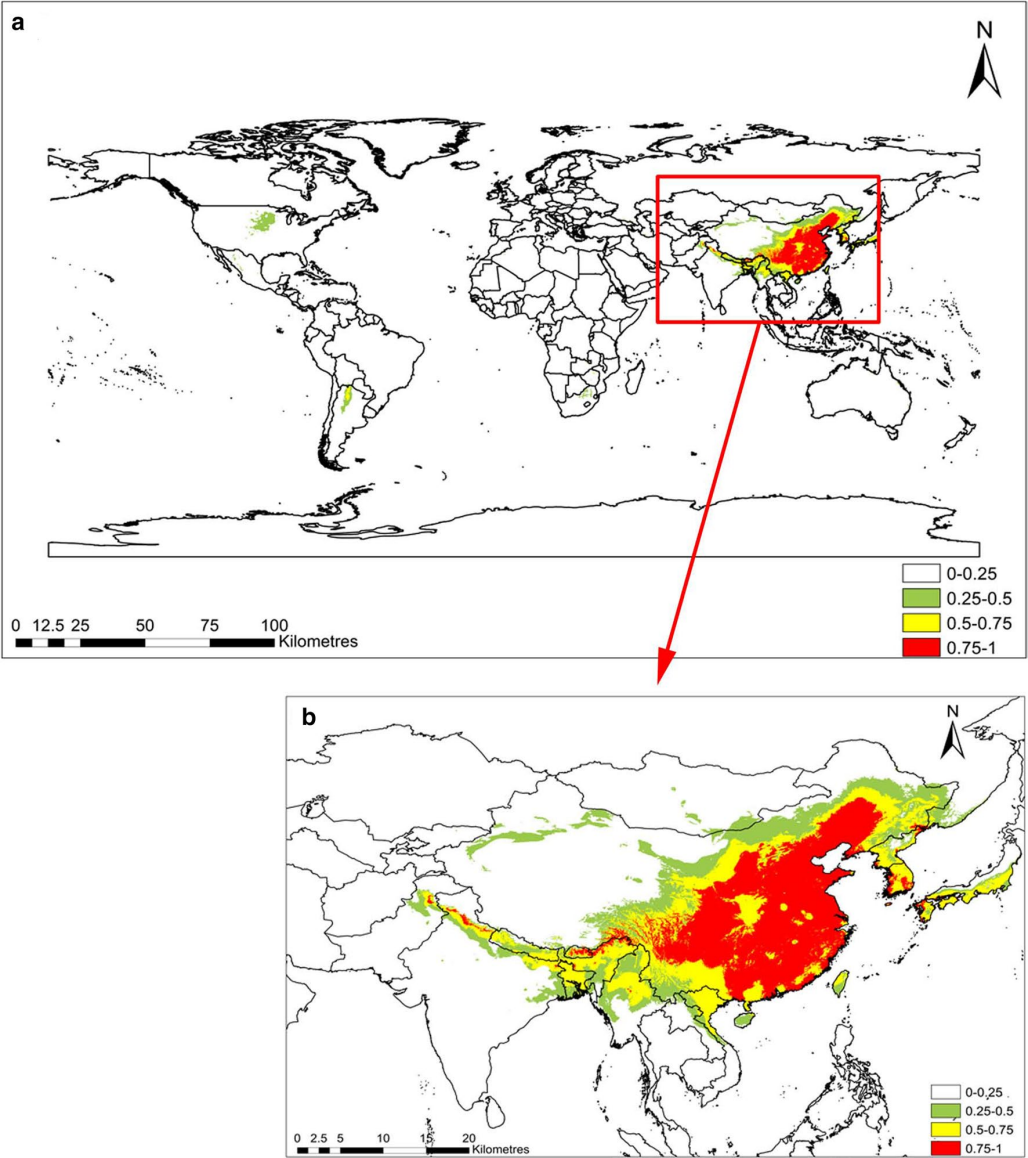
Suitable habitat distribution of *Cuscuta chinensis* in RCP2.6. Geographical base map data were downloaded from the national basic geographic information system (http://www.diva-gis.org/)

**Fig. 4:**
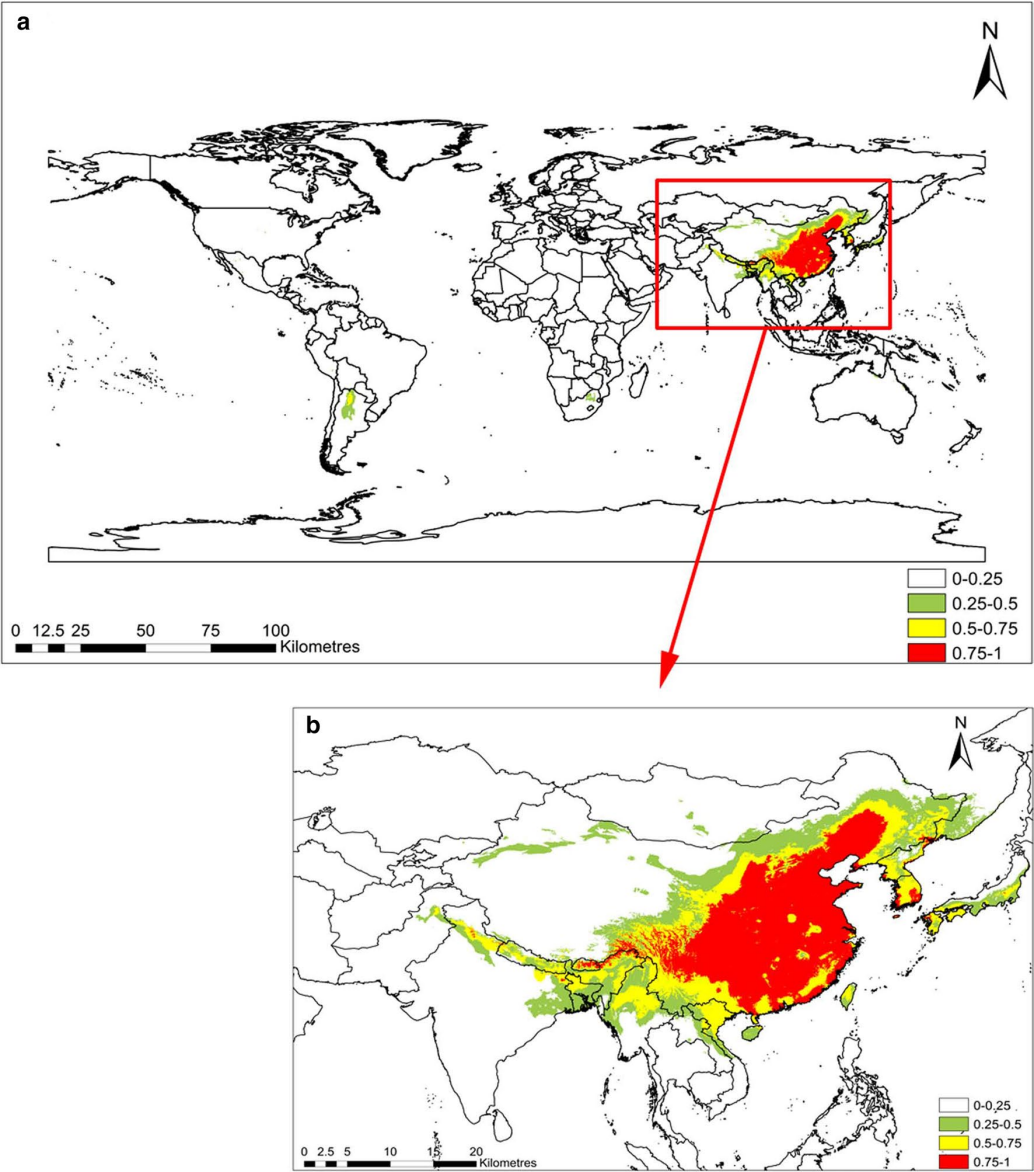
Suitable habitat distribution of *Cuscuta chinensis* in RCP8.5. Geographical base map data were downloaded from the national basic geographic information system (http://www.diva-gis.org/)

The computed results for the *C. chinensis* habitat suitability in RCP2.6 and RCP8.5 are shown in Figs. 3 and 4, respectively. The suitable habitats of *C. chinensis* decreased in response to climatic warming. In RCP8.5, the total area with intermediate suitability and high suitability for the survival of *C. chinensis* was less than that of RCP2.6. In RCP2.6, the *C. chinensis* suitabilities of northern, central, and southern China, North Korea, and the coastal areas of Japan all exceed 0.75. However, the suitabilities of southern Africa, the central and southern parts of North America, and South America ranged between 0.25 and 0.5, while the habitat suitability of the remaining areas was below 0.25. In RCP2.6, the area with suitable habitat was below 0.25 (about 141 million km^2^), accounting for 94.6% of the global area. Areas where the habitat suitability ranged between 0.25 and 0.5, as well as between 0.5 and 0.75 accounted for 2.0% and 1.5% of the world, respectively, with areas of about 2.9526 million km^2^ and 2.2519 million km^2^, respectively. Habitats with suitability exceeding 0.75 accounted for 1.91% of the total area of the world. In RCP8.5, the area suitable for *C. chinensis* growth between 0.25 and 0.5 was the same as in RCP2.6, and its distribution concentrated in the central and southern parts of North America and South America. Moreover, habitats with suitability above 0.75 were also distributed in Northern China, North Korea, and the coastal areas of Japan. Compared with RCP2.6, for RCP8.5, the areas with high suitability for survival increased by 0.052 million km^2^; however, areas with intermediate suitability and high suitability for survival decreased by 0.18 million km^2^. Areas with habitat suitability below 0.25% accounted for 94.8% of the world’s total area, with an area of 141 million km^2^. Compared with RCP2.6, this indicates an increase of 0.3298 million km^2^. Therefore, in general, habitats suitable for *C. chinensis* decreased in response to climate warming (Table 3).

**Table 3:** The area of *Cuscuta chinensis*’s four habitat suitability distribution for two global warming scenarios

Habitat suitability	Global warming scenarios			
	RCP2.6		RCP8.5	
	Area (million km^2^)	PCT (%)	Area (million km^2^)	PCT (%)
0–0.25	140.8488	94.59	141.1786	94.81
0.25–0.5	2.9526	1.98	2.8043	1.88
0.5–0.75	2.2519	1.51	2.0183	1.36
0.75–1	2.8467	1.91	2.8988	1.95

PCT indicates the percentage of the area in the current historical periods of the global’s total area

## Discussion

### Relationship between *C. chinensis* habitat suitability and environmental variables

Temperature and precipitation are two climatic features that can be used as useful starting points to investigate the mechanisms with which the global climate controls plant distribution [29]. Among the six bioclimatic variables adopted for the developed model, annual mean temperature (Bio1), isothermality (Bio3), precipitation seasonality (Bio15), and precipitation of the warmest quarter (Bio18) were the most important contributors to the habitat suitability of *C. chinensis* as indicated by their high weights when used independently. The suitable annual average temperature of *C. chinensis* was identified to range within 11–37 °C. This suitable temperature fits the suitable germination temperature of *C. chinensis*. *C. chinensis* begins to germinate (the germination rate of *C. chinensis* is 7.3%) at a temperature of 10 °C, and the germination rate increases with increasing temperature [30]. When the temperature reaches 25–35 °C, the germination rate of *C. chinensis* reaches 40–46% [30]. When the temperature reaches 40 °C or above, the germination rate of *C. chinensis* is 0 [30]. The isothermality is the mean diurnal range vs. the temperature annual range, which has been shown to affect the distribution of tree species [31]. The distribution of *C. chinensis* is also affected by isothermality. When Bio3 ranged from 0 to 45, the survival probability of *C. chinensis* exceeded 0.5. Precipitation changes during the growing season affect the growth of plants and their primary productivity [32]. In this study, the suitable precipitation of the warmest quarter was 300–1000 mm and 2500–3500 mm for *C. chinensis* growth, indicating that *C. chinensis* prefers a warm and humid environment [33]. *C. chinensis* has a disjunct distribution in Australia and Asia, which is likely the result of its relatively recent long-distance dispersal [28]. The main distribution areas of *C. chinensis* in China include the provinces of Henan, Jiangsu, Shandong, Hebei, Jilin, and Liaoning [56]. Recently, new records of *C. chinensis* have been reported for Bhubaneswar, Odisha, India, and Sikkim [34, 35]. The current distribution of *C. chinensis* might be the result of vegetation changes due to climatic changes, which include temperature and precipitation.

### Habitat suitability simulation for three historical periods

Based on fossil pollen data, Martin predicted that the species in family Convolvulaceae first appeared in the late Eocene of southern Australia, the early Eocene of Africa, or the mid-Eocene of Brazil, and spread from low latitude to high latitude [36]. The results of this prediction indicated that from the last glacial maximum to the mid-Holocene, the global habitat suitability of *C. chinensis* gradually increased. Compared with its current distribution, *C. chinensis* benefitted from a larger area with suitable climate during the mid-Holocene period. Research has shown that the warming and increased precipitation at around 6000 BP greatly affected the European vegetation [37]. As shown in Fig. 2, in Western Europe, the survival probability during the mid-Holocene increased significantly compared with the last glacial maximum. This might be the result of the increased temperature and precipitation during the mid-Holocene, which results in an increase of the suitable survival area of *C. chinensis* [38, 39]. In addition, *C. chinensis* might grow better under the prevailing higher CO_2_ conditions during the mid-Holocene [38, 39]. However, from the mid-Holocene to 1960–1990, the global habitat suitability of *C. chinensis* gradually decreased. The possible reason might be habitat loss, which affects the distribution of species [40]. Meulebrouck et al. reported a significant decline of many wasteland species in Western Europe over the recent decades due to habitat loss [40].

Moreover, *C. chinensis* is a holoparasitic plant, which absorbs both water and nutrients from host plants by haustaria and fully relies on their host [41]. Using Maxent modeling, this study found that the trends of the global habitat suitability of the host (*Glycine max*) of *C. chinensis* during these three historical periods were the same as that mentioned above (unpublished data). This indicates that the habitat suitability of the host would also affect the habitat suitability of the parasite.

### Changes in distribution of *C. chinensis* in the future

From the perspective of the overall ecological suitability of *C. chinensis*, in RCP8.5, the area suitable for the survival of *C. chinensis* was less than that of RCP2.6. Global warming thus seems to have limited the growth of *C. chinensis* and negatively impacted the global habitat suitability and suitable areas for *C. chinensis*. This may be because transitory or constantly high temperatures cause an array of morpho-anatomical, physiological, and biochemical changes in plants. These affect plant growth and development [42], and decrease the productivity of both above-ground plant parts and roots [43].

As mentioned above, the suitability of the host can also affect the future suitability of parasites. Using Maxent modeling, this study found that the suitability area of the host *G. max* in RCP8.5 was less than in RCP2.6 (Ren et al., unpublished data), indicating that global warming also negatively affects the suitable area of the host and consequently reduced the suitable area of parasites by limiting plant growth. In previous literature, several researchers reported that the yield of rice (*Oryza sativa*), chillies (*Capsicum annuum*), and tomato (*Solanum lycopersicon*), other hosts of *C. chinensis* [44], significantly decreased under global warming [45, 46]. This might also reduce the suitability of both hosts and parasites.

As shown in Fig. 4, in the future, *C. chinensis* has a probability of habitat suitability ranging within 0.25–0.5 in both South America and North America under global warming, although *C. chinensis* is currently not distributed there. This might be due to two reasons. First, the temperature and precipitation in South America and North America increased in response to global warming. For example, Ramos da Silva and Haas [47] reported that the overall temperature of South America increased, and precipitation also increased in southern Brazil and the western Amazon in response to global warming. Karmalkar and Bradley [48] showed that the temperature in North America will have increased appropriately by 2050. Projections of the winter precipitation for the eastern USA (including the Northeast) and the Midwest (mostly CMIP5 models) indicate a wetter future. Secondly, with increasing temperature and precipitation, the stronger growth of the *C. chinensis*’s host *Triticum aestivum* [49], a main host of crop in both North and South America [50], might drive the invasion of *C. chinensis* from its suitability area to North America and South America.

## Conclusion

The existence and potentially suitable habitat of *C. chinensis* were assessed and predicted by using the best Maxent modeling evaluated by both the AUC index and TSS index. Six main bioclimatic variables that influence species distribution were selected from a total of 19 bioclimatic variables. These are annual mean temperature, isothermality, temperature seasonality, precipitation seasonality, precipitation of the warmest quarter, and precipitation of the coldest quarter. Controlling temperature and precipitation can both prevent and protect *C. chinensis*. The suitable habitat of *C. chinensis* is mainly distributed in China. Compared with its current distribution, the mid-Holocene period offered a larger climatically suitable area for *C. chinensis*, and central and southern China were particularly suitable. The simulation results of *C. chinensis* habitat suitability in RCP2.6 and RCP8.5 indicated that the *C. chinensis* habitat suitability decreased due to the warming climate. This indicates a decreasing trend for the *C. chinensis* distribution in the future. In addition to the above bioclimatic variables, other factors may also affect the suitable habitat of plants, such as soil, geographic barriers, human disturbance, and host distribution [41, 42]. Although this study only considered the impact of the climate on *C. chinensis*, if the effects of human activities, geographic barriers, soil conditions on vegetation, and host distribution were to be comprehensively considered, the distribution of *C. chinensis* could be more accurately predicted. However, a flaw affects the accuracy of ENMs, which critically hinges on the quality of the occurrence data and often uses haphazardly collected data. Although the maximum number of background points was set to 10,000, to match the bias of the buffer of appearance records, the utilized background records were constrained to Asia.

## Methods

### Study species

*Cuscuta chinensis* grows near the seaside, its stems are thin, twining, filiform, glabrous, yellowish or pale yellowish, and have a diameter of ~ 1 mm (Fig. 1b). The plant has neither roots nor leaves, or leaves that are reduced to minute scales [51]. It often parasitizes on Fabaceae, Asteraceae, or Zygophyllaceae. *C. chinensis* is distributed throughout Asia and Australia [51, 52]. Its aerial parts are harvested in autumn, when the fruits are ripe, and are dried naturally via sunlight. Dried parts are thrashed for seeds [28, 51]. *C. chinensis* seeds are often used as herbal medicine, and have the functions to improve the metabolism of the liver and kidney, are used as a diuretic, and can improve eyesight [15, 53, 54].

### Data sources

Occurrence records (818) of *C. chinensis* were collected from the national specimen information infrastructure (http://www.nsii.org.cn/), the Chinese virtual herbarium (http://www.cvh.org.cn/), and the plant photo bank of China (http://ppbc.iplant.cn/). 175 occurrence records were collected from the global biodiversity information facility (GBIF; http://www.gbif.org; accessed on June 26, 2018). Moreover, four occurrence records were collected from scientific publications. The total records were also filtered at the resolution of 2.5 arc min (4.3 × 4.3 km^2^) and 550 similar-latitude and longitude-repeated records as well as four invalid records (occurrence points of the bioclimatic variable raster layer with a value of -9999) were deleted. Finally, 443 valid records were used for the analysis herein (Additional file 3: Table S1).

Bioclimatic factors exert important biological significance for the determination of the environmental niche of species [2]. Since the GBIF database has a spatially-biased dataset due to variable sampling efforts, data storage, and mobilization below 30 arc sec resolution [55, 56], only data of 19 bioclimatic factors with 2.5 (4.3 × 4.3 km^2^), 5 (10 × 10 km^2^), and 10 arc min (16 × 16 km^2^) resolution were downloaded (http://www.worldclim.org). The climate data for the three periods (last glacial maximum, Mid-Holocene, and 1960–1990) also originate from http://www.worldclim.org.

The paleoclimatic prediction was conducted with the CCSM4 climate model. The representative concentration pathways (RCPs) are four greenhouse gas concentration (rather than emission) trajectories that have been adopted by the Intergovernmental Panel on Climate Change (IPCC) [4]. Four RCPs (RCP2.6, RCP4.5, RCP6.0, and RCP8.5) represent net radiative forcing of 2.6, 4.5, 6.0, and 8.5 W/m^2^ at the end of the year 2100 [57, 58]. The most representative RCP2.6 and RCP8.5 with the lowest and highest net radiation intensity at the end of 2100, respectively, were used for this study. The RCP2.6 scenario results in 490 ppm CO_2_ equivalent and a global average temperature increase of 1.5 °C, while the RCP8.5 scenario results in 1370 ppm CO_2_ equivalent and a global average temperature increase of 5.0 °C [59, 60]. *C. chinensis* habitat suitability distributions were modeled for each of these two scenarios. In both cases, the habitat suitability distribution of *C. chinensis* was simulated separately. Climate projections for the years 2061 through to 2080 were used as climate data and were obtained from CCSM4 global climate models for RCP2.6 and RCP8.5, which are available at http://www.worldclim.org. Geographical base map data were downloaded from the national basic geographic information system (http://www.diva-gis.org/).

### Bioclimatic variable screening

To avoid the influence of highly relevant environmental data on the prediction results, both Pearson correlation coefficient and principal component analysis (PCA) of 19 bioclimatic variables were tested using the SPSS 19.0 software (SPSS Inc. Chicago, IL USA). One variable of each set of highly cross-correlated variables (r > 0.8) was selected for further analysis [2]. Various environmental factors were considered and the most relevant factors for prediction and evaluation were selected [61, 62]. Subsequently, the Maxent model was used to calculate the contribution rate of the 19 selected environmental factors. According to the 19 bioclimatic variables presented in Table 4, 10% of the distributed information points were randomly selected as test sets, and the remaining 90% of the samples were used as training set for model verification. The model settings were repeated 10 times. Six bioclimatic variables were screened to explore the response of *C. chinensis* to climate change. Then, the Jackknife test was used to test the contribution rate of bioclimatically dominant factors (> 0.4).

**Table 4:** Bioclimatic variables used in the model and the relative contributions of 19 bioclimatic variables to the Maxent model for *C. chinensis*

Variables	Description	Percent contribution
BIO1	Annual mean temperature	8.6
BIO2	Mean diurnal range (Mean of monthly (max temp − min temp))	1.8
BIO3	Isothermality (BIO2/BIO7) (* 100)	6.4
BIO4	Temperature seasonality (standard deviation * 100)	13.1
BIO5	Max temperature of warmest month	1.4
BIO6	Min temperature of coldest month	1.8
BIO7	Temperature annual range (BIO5–BIO6)	1.3
BIO8	Mean temperature of wettest quarter	1.1
BIO9	Mean temperature of driest quarter	3.9
BIO10	Mean temperature of warmest quarter	2
BIO11	Mean temperature of coldest quarter	3.6
BIO12	Annual precipitation	1.6
BIO13	Precipitation of wettest month	8
BIO14	Precipitation of driest month	1.2
BIO15	Precipitation seasonality (coefficient of variation)	13.4
BIO16	Precipitation of wettest quarter	1.3
BIO17	Precipitation of driest quarter	1.2
BIO18	Precipitation of warmest quarter	24.1
BIO19	Precipitation of coldest quarter	4.2

### Maxent modelling

The principle of Maxent is a criterion for the selection of statistical characteristics of random variables that best meet the objective conditions. This is also known as the principle of maximum information. The probability distribution of random quantities is difficult to measure. Generally, only mean values (such as the mathematical expectation and variance) or values under specific defined conditions (such as peak values and the number of values) can be measured. The distribution of these values can be measured in a variety of ways, and thus, an infinite number of distributions can be investigated. Typically, one of these distributions has the highest entropy. Selecting this distribution with the highest entropy (Maxent) as distribution of the random variable is an effective processing method and criterion. This Maxent approach establishes a model with Maxent in accordance with known data [23, 41]. Maxent uses all grid elements in a certain study area, thus using the largest possible distribution space. It also uses the grid unit of the known species distribution point as sample point, and obtains the constraining conditions according to the environmental variables of the sample point unit to identify the Maxent under this constraint condition. This approach yields the possible distribution [23, 63] with simple operation, fast calculation speed, and good prediction result [64, 65]. Based on the Maxent theory, the Java-based software package Maxent was developed by Phillips et al. [66] and can be used to simulate habitat suitability. The present study used Maxent modelling to predict the potential distribution of *C. chinensis*. Maxent software (version 3.4.1) was obtained from the official website (http://biodiversityinformatics.amnh.org/opensource/maxent/) [67]. The Maxent method is to establish a model with a maximum entropy in accordance with known knowledge [2]. The entropy of a random variable ξ is given by the formula [2]: $H\left( \xi \right) = \mathop \sum \nolimits_{i = 1}^{n} \left( {p_{i} \log \frac{1}{{p_{i} }}} \right)$.

### Model evaluation index

The area under curve (AUC) value and the true skill statistic (TSS) index respond differently to distribution point occurrence rates and thresholds; therefore, their combination can better assess the performance of the model [68, 69]. Both AUC and TSS were used to evaluate the performance of the model [68]. The AUC value was directly obtained after running the Maxent software [68]. The receiver operating characteristic (ROC) curve is based on the accuracy of the threshold-independent evaluation model, i.e., each value of the prediction result is used as possible judgment threshold. The corresponding sensitivity and specificity were calculated via the ROC curve. The specificity (1-specificity; i.e., the probability for being predicted to be positive without the species distribution) is shown on the abscissa, and the sensitivity (1-omission rate; i.e., the probability that the species is actually distributed and predicted to be positive) is shown on the ordinate. The AUC value was calculated as the area enclosed by the curve and the abscissa and was used to assess the performance of models that are not affected by the choice of the threshold [70]. In general, the AUC ranges between 0.5 and 1. A larger AUC value indicates better model performance [2]. Model performance is categorized as failing (0.5–0.6), poor (0.6–0.7), fair (0.7–0.8), good (0.8–0.9), or excellent (0.9–1) according to the AUC [69]. The TSS provides a threshold-dependent measure of accuracy, which is often applied for presence–absence predictions [68]. The TSS index was calculated as: TSS = Sensitivity + Specificity − 1, where sensitivity is defined as the probability that a model correctly classifies the presence data, whereas specificity indicates the probability of classifying correctly the absence data points [71]. The TSS ranges from − 1 to 1, where a value of 0 or less indicates a model performance no better than random, and a value of 1 indicates perfect performance [71]. Here, model performance is either categorized as failing (< 0.4), poor (0.4–0.55), fair (0.55–0.7), good (0.7–0.85), or excellent (0.85–1) according to the TSS [68].

### Model setting

This study randomly established 10,000 validation sites, and used the actual point of existence to run the model. To obtain the best model, the Maxent model was set as follows: (1) the regularization multiplier (beta) selected based on corrected Akaike information criterion (AICc) was set to 1, 2, 5, 10, 15, and 20 [19, 72, 73]. (2) A 10 cross-validation approach was used as replicated run type [22]. (3) A complementary log–log (cloglog) transformation was used to produce an estimate of the habitat suitability of weeds [67]. (4) The resolutions of environmental variables were set to 2.5, 5.0, and 10.0 arc min. The Maxent model was run 10 times repeatedly. Each run randomly selected 90% of the distribution information points as training set, while the remaining 10% of the samples were used for the test. The threshold rule selected equal training sensitivity and specificity. The output format of Maxent was selected automatically depending on the particular sample size of the occurrence records according to a method developed by Phillips and Dudik [62]. The auto feature setting was selected and the regularization multiplier (beta) was 1. In addition, the environmental variable was set to 2.5 min.

### Predicting the suitable area of *C. chinensis* under global climate change

ArcGIS 10.2 software (ESRI Inc., Redlands, CA, USA) was used to superimpose and map the results of the Maxent model calculations. Based on the main ecological factors, a global map of ecologically appropriate zones for *C. chinensis* was drawn. Artificial grading was used to classify different grades based on their ecological similarity. According to the statistical principle, the expression of probability “existence” and the empirical method divided the suitable area into four levels [74]. These levels are: < 25%, which is representative for non-suitable survival areas, and indicates that the suitability for the survival of species in these areas is below 25%. A level of 25–50% is representative for low-suitable survival areas, a level of 50–75% is representative for survival areas with average suitability, and a level of > 75% is representative for high-suitable for survival area. These three levels indicate that species have a higher probability of survival in these areas [74]. The optimal ranges of the climatic variables were defined as those ranges where species inhabited survival areas with average and high suitability, i.e. > 50% [74]. The range of appropriate eco-factor values was derived from the response curves of the Maxent model results.

## Supplementary information

**Additional file 1: Fig. S1.** The results of the jackknife test of variable contribution in modeling the habitat distribution of *Cuscuta chinensis*. The regularized training gain describes how much better the Maxent distribution fits the data compared to a uniform distribution. The dark blue bars indicate the gains from using each variable in isolation, while the light blue bars indicate the gains lost by removing a single variable from the full model. The red bar indicates the gains when all variables are used.

**Additional file 2: Fig. S2.** Response curves of six main bioclimatic variables. The red curve showed the mean response calculated over 10 replicates, while the blue margin showed the standard deviation calculated over 10 replicates. The temperature data are expressed in  °C * 10. This means that the value of 231 represents 23.1 °C. The unit used for precipitation data is mm (Bio1: annual mean temperature (°C); Bio3: isothermality (BIO2/BIO7) (* 100); Bio4: temperature seasonality (standard deviation *100); Bio15: precipitation seasonality (coefficient of variation); Bio18: precipitation of warmest quarter (mm); Bio19: precipitation of coldest quarter (mm)).

**Additional file 3: Table S1.** 443 valid records used for this study.

## Data Availability

All data generated/used by this study is present in the supplementary material.

## Abbreviations

AUC: Area under curve
ENMs: Ecological niche models
GBIF: Global biodiversity information facility
IPCC: Intergovernmental panel on climate change
PCA: Principal component analysis
RCPs: Representative concentration pathways
ROC: Receiver operating characteristic
TSS: True skill statistic
