# Supplementary figures and images for: Predicting the potential distribution of the parasitic Cuscuta chinensis under global warming
Source: BMC Ecol. 2020 May 9;20:28. doi: 10.1186/s12898-020-00295-6 (PMC7210669; doi:10.1186/s12898-020-00295-6)

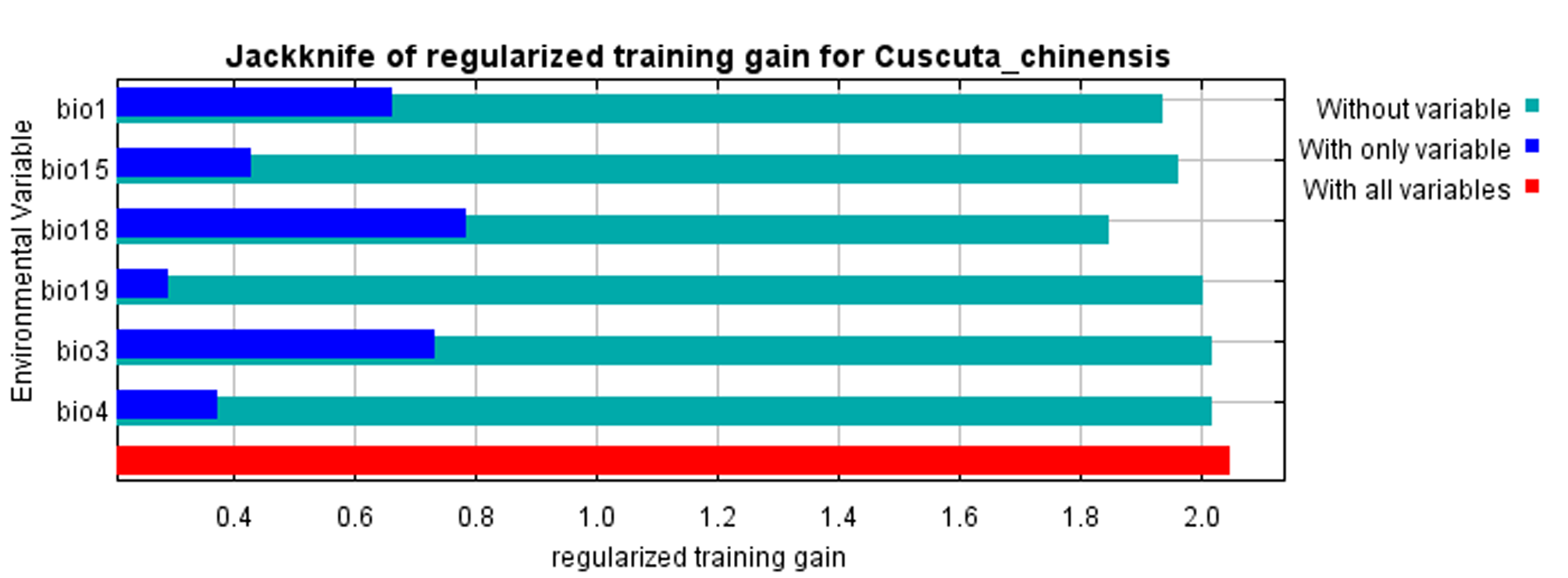

Supplement: Supplementary file 1 — Additional file 1: Fig. S1. The results of the jackknife test of variable contribution in modeling the habitat distribution of Cuscuta chinensis. The regularized training gain describes how much better the Maxent distribution fits the data compared to a uniform distribution. The dark blue bars indicate the gains from using each variable in isolation, while the light blue bars indicate the gains lost by removing a single variable from the full model. The red bar indicates the gains when all variables are used. [file 12898_2020_295_MOESM1_ESM.tif]

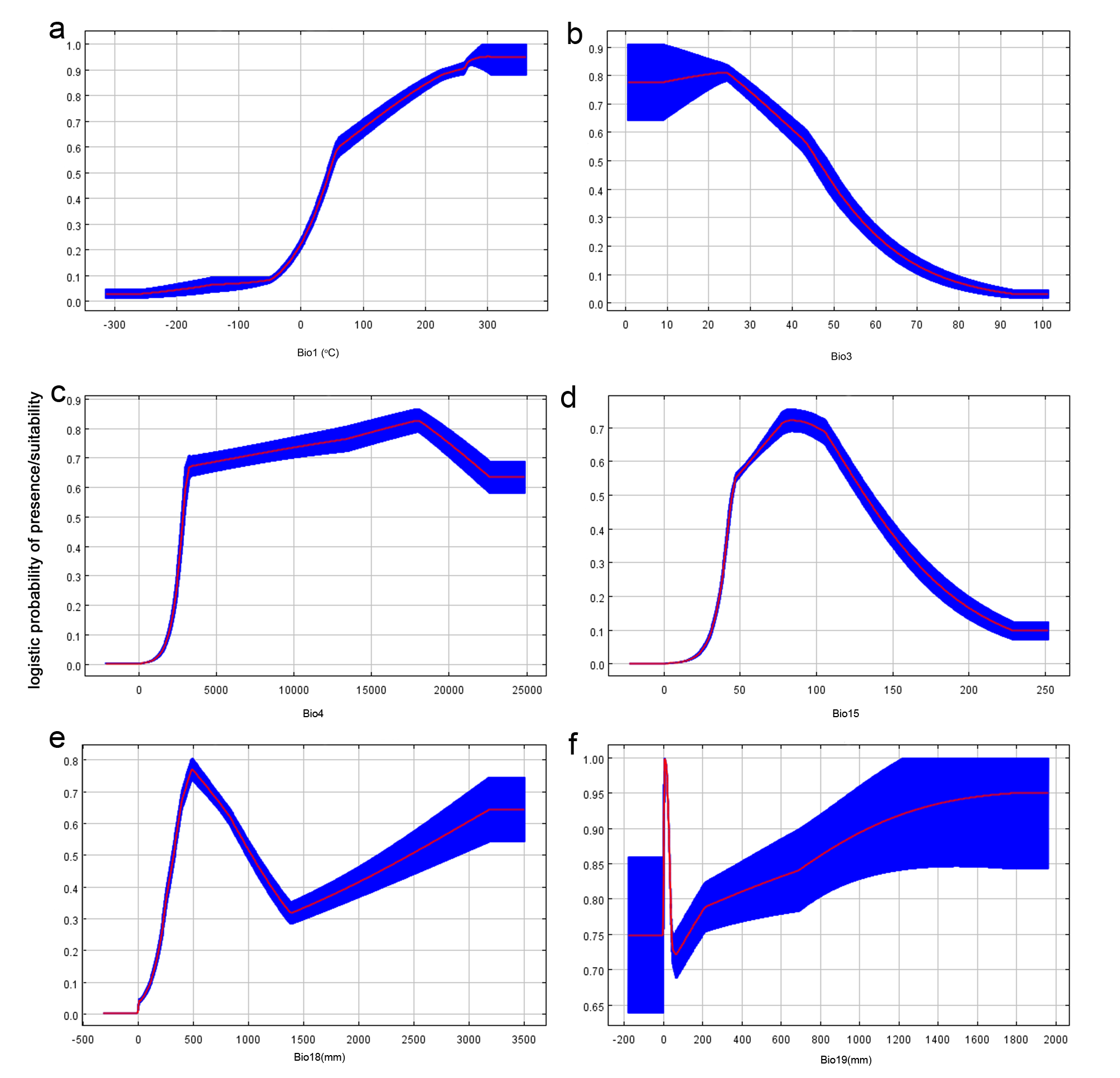

Supplement: Supplementary file 2 — Additional file 2: Fig. S2. Response curves of six main bioclimatic variables. The red curve showed the mean response calculated over 10 replicates, while the blue margin showed the standard deviation calculated over 10 replicates. The temperature data are expressed in °C * 10. This means that the value of 231 represents 23.1 °C. The unit used for precipitation data is mm (Bio1: annual mean temperature (°C); Bio3: isothermality (BIO2/BIO7) (* 100); Bio4: temperature seasonality (standard deviation *100); Bio15: precipitation seasonality (coefficient of variation); Bio18: precipitation of warmest quarter (mm); Bio19: precipitation of coldest quarter (mm)). [file 12898_2020_295_MOESM2_ESM.tif]
